# Molecular Characterization of Barley 3H Semi-Dwarf Genes

**DOI:** 10.1371/journal.pone.0120558

**Published:** 2015-03-31

**Authors:** Haobing Li, Guangdeng Chen, Wei Yan

**Affiliations:** 1 Tasmanian Institute of Agricultural Research and School of Agricultural Science, University of Tasmania, Hobart, Australia; 2 CSIRO Agriculture Flagship, Queensland Bioscience Precinct, St Lucia, Queensland, Australia; 3 Bioscience Research Division, Department of Environment and Primary Industries, Horsham, Victoria, Australia; 4 Institute of Ecological and Environmental Sciences, Sichuan Agricultural University, Wenjiang, Chengdu, China; 5 Institute of Food Crops, Jiangsu Academy of Agricultural Science, Nanjing, China; Mahatma Phule Agricultural University, INDIA

## Abstract

The barley chromosome 3H accommodates many semi-dwarfing genes. To characterize these genes, the two-rowed semi-dwarf Chinese barley landrace ‘TX9425’ was crossed with the Australian barley variety ‘Franklin’ to generate a doubled haploid (DH) population, and major QTLs controlling plant height have been identified in our previous study. The major QTL derived from ‘TX9425’ was targeted to investigate the allelism of the semi-dwarf gene *uzu* in barley. Twelve sets of near-isogenic lines and a large NILF_2_ fine mapping population segregating only for the dwarfing gene from ‘TX9425’ were developed. The semi-dwarfing gene in ‘TX9425’ was located within a 2.8 cM region close to the centromere on chromosome 3H by fine mapping. Molecular cloning and sequence analyses showed that the ‘TX9425’-derived allele contained a single nucleotide substitution from A to G at position 2612 of the *HvBRI1* gene. This was apparently the same mutation as that reported in six-rowed *uzu* barley. Markers co-segregating with the QTL were developed from the sequence of the *HvBRI1* gene and were validated in the ‘TX9425’/‘Franklin’ DH population. The other major dwarfing QTL derived from the Franklin variety was distally located on chromosome 3HL and co-segregated with the *sdw*1 diagnostic marker *hv20ox2*. A third dwarfing gene, expressed only in winter-sown trials, was identified and located on chromosome 3HS. The effects and interactions of these dwarfing genes under different growing conditions are discussed. These results improve our understanding of the genetic mechanisms controlling semi-dwarf stature in barley and provide diagnostic markers for the selection of semi-dwarfness in barley breeding programs.

## Introduction

Semi-dwarf genes have been thoroughly explored in cereal crops, including the *sd1* semi-dwarf gene in rice [1, 2] and the *Rht* genes in wheat [3], which contributed to the success of the Green Revolution. Several short-statured genotypes have been identified in barley [4, 5], and genotypes possessing the semi-dwarf genes *uzu* and *sdw1* have been widely used in breeding programs to reduce lodging and to improve the harvest index.

The *uzu* semi-dwarf barley varieties are commonly cultivated in China, Japan, and the Korean peninsula. Nearly 80% of the 147 Chinese short-strawed barley varieties contain the *uzu* dwarf gene [6, 7]. In the 1930s, *uzu* varieties occupied 70% of the barley culture areas in Japan and more than 30% of such areas in the Korean peninsula [8]; the *uzu* gene is currently being introduced into all hull-less barley varieties cultivated in Japan [9]. Extensive studies of the underlying morphological, physiological, and molecular mechanisms have shown that semi-dwarf barley accessions carrying the *uzu* gene do not respond to brassinosteroids (BRs), which are essential plant hormones that play important roles in various aspects of plant growth and development, including germination, cell elongation, growth and flowering [10]. A synteny study suggested the *uzu* gene to be homologous to the rice gene *D61*. The latter is a homolog of the *Arabidopsis thaliana* BR-insensitive1 gene (*BRI1*), which encodes a plasma membrane-bound BR-receptor protein that is involved in cell wall expansion preceding cell elongation in living cells of *Arabidopsis* seedlings [11]. A barley homologue of *BRI1* (i.e., *HvBRI1*) was isolated from a six-rowed *uzu* barley genotype, and a sequence analysis showed that the *uzu* phenotype may be caused by a single-nucleotide substitution (A to G) at position 2612 of *HvBRI1* [9]. This mutation results in an amino acid change at a highly conserved residue (His-857 to Arg-857) of the kinase domain of the BRI1 receptor protein, leading to reduced sensitivity to BRs and reduced plant height [9]. The *HvBRI1* gene was mapped to the barley genome, using a six-rowed F2 population segregating for *uzu* [9]. Co-segregation of the single nucleotide polymorphism (SNP) and the *uzu* trait was further determined in 263 *uzu* and 55 normal barley lines originating from East Asia [12].

Previous *uzu*-related research has focused on the *uzu* allele originating from the six-rowed barley germplasm [12] because spontaneous two-rowed *uzu* barley genotypes are very rare. However, genetic diversity studies based on molecular markers have divided the barley germplasm into two different categories, suggesting that two-rowed (*distichon* var.) and six-rowed (*hexastichon* var.) barley are genetically distant and probably have different origins [13, 14]. As independent mutations have been reported in the same semi-dwarf genes in rice and wheat [2, 15], the *uzu* genes/alleles in the barley germplasm must be investigated, especially in two-rowed barley landraces originating from East Asia.

The barley semi-dwarf gene *sdw*1 has been reported as being allelic to the *denso* gene, although they were derived from different sources. The *sdw1* gene originated from the Norwegian ‘Jotun’ variety, and *denso* from the Danish ‘Abed Denso’ variety [16]. The *sdw1* barley mutant has been widely used to develop short-statured cultivars for feed production in the Western USA, Canada, and Australia [17, 18]. In contrast, the *denso* gene has gained wide acceptance in European malting barley [17, 19–22]. Both *sdw1-* and *denso-*containing barleys have similar agronomic traits, such as late heading, low seed weight, low yield, and high screening [17, 21]. However, some *sdw*1-carrying varieties, such as feed barley UC 828 [23], display increased grain yield, better grain size, and low screening. Jia et al. [24] have found that *sdw1* in barley is the most likely ortholog of *sd1* in rice, and GA-20 oxidase has been identified as a candidate for the semi-dwarf gene *sdw1*/*denso* in barley.

Although *sdw*1/*denso* and *uzu* are all located on barley chromosome 3H [20], *sdw*1 is sensitive to gibberellin (GA), whereas *uzu* is not. Therefore, it would be interesting and useful to investigate the effect and interaction of these genes/alleles in the same genetic population, and to provide breeders with the precise genomic locations of these genes and with closely linked markers for marker-assisted selection (MAS). In the present study, we report the fine-mapping and molecular cloning of a semi-dwarf gene derived from a two-rowed barley landrace variety, the characterization of other genes on chromosome 3H, and the development and validation of molecular markers co-segregating with these genes.

## Materials and Methods

### Development and assessment of near-isogenic lines for the dwarf locus in the Chinese two-rowed landrace ‘TX9425’

The two-rowed dwarf barley landrace ‘TX9425’ shows some characteristics that are typical of so-called *uzu* barley, such as the unique elongation of coleoptiles, leaves, culms, rachis internodes, awns, glumes, and kernels. Under spring sown conditions, coleoptiles of ‘TX9425’ show a prominent projection or hook near the apex, and sometimes a V-shaped notch on the opposite side.

A doubled haploid (DH) population with 120 lines was developed from a cross between ‘TX9425’ and Australian variety ‘Franklin’. A linkage map was constructed for this population by using 412 Diversity Array Technology (DArT) markers, 80 amplified fragment length polymorphism (AFLP) markers, and 28 microsatellite markers [25]. The SSR markers HVM33 and Bmac0209 were found flanking the major semi-dwarf QTL derived from ‘TX9425’ [26].

The heterogeneous inbred family (HIF) method [27] was used to develop near-isogenic lines (NILs) for the ‘TX9425’ semi-dwarf locus from an F_2_ ‘TX9425/Franklin’ population. The co-dominant simple sequence repeat (SSR) marker HVM33, which was most closely linked to the peak of the QTL controlling plant height [26], was used to select F_2_ individuals that were heterozygous at the marker locus. The selection followed the method described by Ma et al. [28]. Briefly, 12 individual heterozygous F_2_ plants were identified and self-pollinated. Six F_3_ plants were grown from each heterozygous F_2_ plant, and a single heterozygote was again selected and self-pollinated. This process of heterozygous individual selection followed by self-pollination was repeated until the F_8_ generation and produced a total of 269 plants. From each of the 12 original F_2_ plants, two homozygous F_8_ lines, one with and the other without the ‘TX9425’ allele, were selected and were treated as a pair of NILs.

The NILs and the two parents were assessed in two trials: the first was a pot trial performed in a greenhouse, and the second was a field trial conducted at the CSIRO Research Station at Gatton, Queensland, Australia (27°34′S, 152°20′E). The pot trial was conducted using six plants per line, with three plants in each of two 2-L pots, using a completely randomized design. Plant height measurements were made on the two tallest tillers on each plant, and the mean value was used in subsequent statistical analyses. Two replicates of the field trial were sown in June 2011. Twenty seeds of each NIL were grown in a single 1.5 m row in each replicate, the space between rows was 25 cm. Measurements were taken on the six tallest tillers in each row, and the average values were used in subsequent statistical analyses.

### Development and assessment of a NILF2 population segregating only for the target semi-dwarf gene derived from ‘TX9425’

Three ‘TX9425/Franklin’ F_8_ plants, presumably heterozygous only at the HVM33 marker locus and homologous at all other genome regions, were used to generate a population segregating only for the target semi-dwarf locus derived from ‘TX9425’. F_9_ seeds from the three plants were combined to generate a population with 903 individual plants. This large mapping population was equivalent to an F_2_ population, and was named NILF_2_. Each NILF_2_ plant was grown in a 2L pot in a greenhouse, and its height was assessed based on the mean of the three tallest tillers.

### Fine mapping of the dwarfing gene derived from ‘TX9425’

The NILF_2_ population was used in the fine mapping of the dwarfing gene derived from ‘TX9425’. Thirty-two polymorphic PCR-based markers adjacent to this region were used to screen a subset of 192 NILF_2_ lines. A linkage map was constructed using these NILF_2_ plants, and the markers flanking the dwarf locus identified in the subset were genotyped in the NILF_2_ plants remaining in the fine mapping population. The quantitative height values of the 903 NILF_2_ plants were converted to qualitative data, based on which of the two height groups they belonged to. These quantitative height data were integrated to the molecular genotypic data of genetic linkage mapping using the JoinMap 4.0 software [29] to locate the position of the dwarfing gene on barley chromosome 3H.

### Molecular cloning and sequencing of the target semi-dwarfing gene

A pair of NILs segregating for the semi-dwarf gene was grown in a mixture of soil and vermiculite (1:1) in a growth chamber at 18°C under a 16 h photoperiod and 200 μEm^-2^s^-1^ light intensity. Four weeks after planting, bulked leaf tissue samples from five plants in each line were collected for DNA extraction, which was performed according to the “micro C-TAB” protocol [30].

Six primer pairs (Table 1) were used to clone the *HvBRI1* gene. Three independent “touch-down” PCRs were performed per primer pair for each genotype. The PCR reaction mixture (20 μL) comprised 1 μL of DNA of a given genotype (0.1 μg/μL), 10 mM of Tris-HCl, 1.5 mM of MgCl_2_, 50 mM of KCl, 0.1% Triton X-100, 0.2 mM of dNTPs, 10 pM of forward primer, 10 pM of reverse primer, 0.7 units of *Taq* polymerase, and distilled water added to achieve the final volume. The following PCR amplification profile was performed for primer pairs 2, 3, and 5: (1) 94°C, 3 min; (2) 94°C, 45 s; (3) 62°C, 45 s; (4) 72°C, 1 min 30 s; steps 2–4 repeated 3 times; (5) 94°C, 45 s; (6) 60°C, 45 s; (7) 72°C, 1 min 30 s; steps 5–7 repeated 3 times; (8) 94°C, 45 s; (9) 57.5°C, 45 s; (10) 72°C, 1 min 30 s; steps 8–10 repeated 32 times; and (11) final extension at 72°C, 5 min. For primer pairs 1, 4, and 6, the following PCR profile was performedused: (1) 94°C, 3 min; (2) 94°C, 45 s; (3) 59°C, 45 s; (4) 72°C, 1 min 30 s; steps 2–4 repeated 3 times; (5) 94°C, 45 s; (6) 57°C, 45 s; (7) 72°C, 1 min 30 s; steps 5–7 repeated 3 times; (8) 94°C, 45 s; (9) 55°C, 45 s; (10) 72°C, 1 min 30 s; steps 8–10 repeated 32 times; and (11) final extension at 72°C, 5 min.

**Table 1:** Primers used to clone *HvBRI1* in NIL lines segregating for the ‘TX9425’ dwarfing locus.

Primer name	Forward primer (5′–3′)	Reverse primer (5′–3′)	Annealing temperature (°C)	Reference
P-uzu1	CGCTTCTCGCATGGTCTC	CAGCGAAGGTCGGCATCT	49.5	Developed in this study
P-uzu2	CTCGACTTGTCCAGCAACAA	GTTGGGATCTTGGCAGAGG	57.5	Gruszka et al. (2011)
P-uzu3	CGACCTCAGCTCCAACAACT	TCCTTGTGAAGTTGCACAGC	55	Gruszka et al. (2011)
P-uzu4	CTGAGCAGCCAGTGTCGT	TACTTGCCTCGTCATTCTTCT	55	Developed in this study
P-uzu5	GGAGGCAGAAGAATGACGAG	CAGCAACACAACACCGTAGC	57.5	Gruszka et al. (2011)
P-uzu6	GGTATGTGCCACCGGAGTA	AGCTTGCGTGGGAACCTCA	55	Gruszka et al., (2011)

Amplification products were extracted from 1% agarose gels using a QIAquick Extraction Kit (Qiagen) and cloned into a TOPO vector (Invitrogen). The transformed, competent *Escherichia coli* cells were incubated overnight at 37°C on an LB agar plate containing the Blue-White Select Screening Reagent (Sigma). Three white colonies for each insert were chosen, and transferred to a liquid LB medium for plasmid isolation as sequencing controls. Plasmid purification was performed using a QIAprep Spin Miniprep Kit (Qiagen). Inserts were sequenced by the Australian Genome Research Facility Ltd. (Queensland). The analysis of the sequencing data was performed using the AlignIR software (LI-COR, Lincoln, NE, USA), which allowed the assembly and alignment of sequences derived from different genotypes, to identify mutations.

To detect SNPs in the semi-dwarf gene from ‘TX9425’, the derived cleaved amplified polymorphic sequence (dCAPS) method was performed, as described by Michaels and Amasino [31] as well as Saisho et al. [12]. Genomic DNA from semi-dwarf and tall NILs was used as a PCR template, and the forward primer 5′-GAAATGGAGACCATTGGCAAGATCAAGC-3′ and reverse primer 5′-CCTTGCCTCCAGATTCTCATCAAC-3′ were used. The restriction enzyme *Hha*I was used for the detection of SNPs in the target semi-dwarfing gene.

### Validation of closely linked markers of different semi-dwarfing genes identified on the ‘TX9425/Franklin’ background

In order to validate and characterize the semi-dwarfing genes on barley chromosome 3H, three pairs of CAP primers were designed from the ‘TX9425’ dwarfing gene sequence. Markers developed from the semi-dwarf gene derived from ‘TX9425’ and the *sdw*1 diagnostic marker of Hv20ox2 [24] were used to genotype a ‘TX9425/Franklin’ DH population to confirm the association of these diagnostic markers with QTLs controlling plant height in this population. Linkage and QTL analyses were applied using the Joinmap_4.0 and MapQTL_5.0 softwares [32], as described by Li et al. [26]. The locations of these diagnostic markers were also compared to those in the barley bin map published on the GrainGenes website (http://wheat.pw.usda.gov) to further confirm the locations of these genes in the barley genome.

## Results

### Morphological characteristics of the populations derived from ‘TX9425/Franklin’

**Fig 1:**
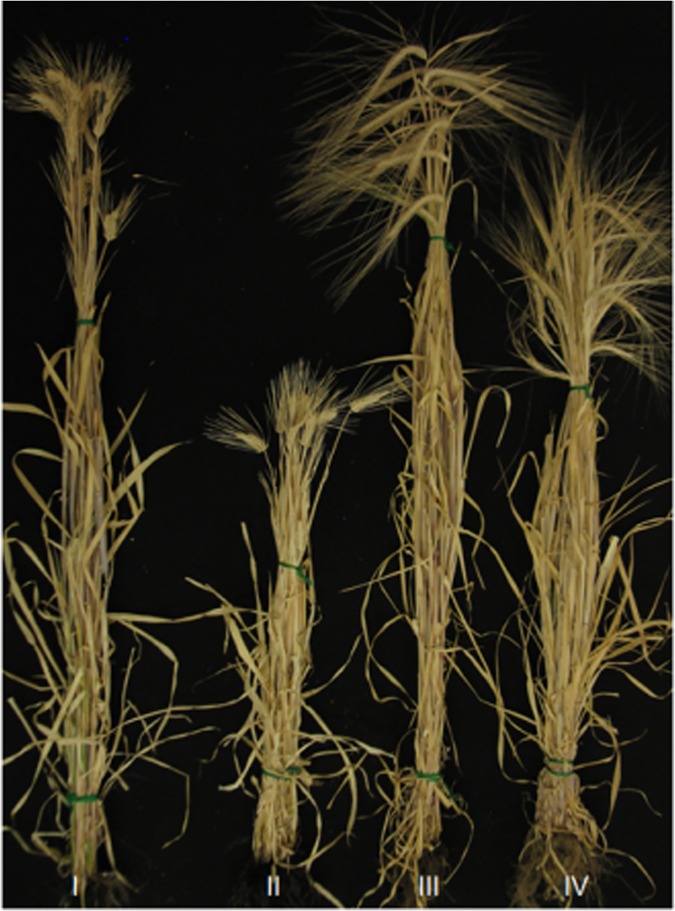
Phenotypes and height of the short-statured barley genotypes ‘TX9425’ and ‘Franklin’ grown under different temperature and day length conditions. (I) ‘TX9425’, grown in low temperatures and during short days; (II) ‘TX9425’, grown in warm temperatures and during long days; (III) ‘Franklin’, grown in low temperatures and during short days; and (IV) ‘Franklin’, grown in warm temperatures and during long days.

The growth of the parental line ‘TX9425’ was significantly affected by the environments, and their height under spring-sown conditions was less than two-thirds of that following winter planting (Fig. 1). The two parental genotypes led to similar plant heights when they were grown in the field in low temperatures and short days following winter planting (Table 2; Fig. 1). However, when they were grown in warm temperatures and during long days, ‘TX9425’ plants were 33–50% shorter than ‘Franklin’ plants.

**Table 2:** Distribution of plant height (cm) in the doubled haploid and NILF2 populations.

Population	Mean for parents		Lines			
	‘TX9425’	‘Franklin’	Minimum	Maximum	Mean	SD
‘TX9425/Franklin’ DH (winter-sown)	110.00	115.00	58.00	143.00	107.00	18.00
‘TX9425/Franklin’ DH (spring-sown)	36.64	83.40	25.25	83.75	42.95	13.72
‘TX9425/Franklin’ NILF2 (fine-mapping population)	57.60	102.70	30.00	130.00	98.05	12.58

**Fig 2:**
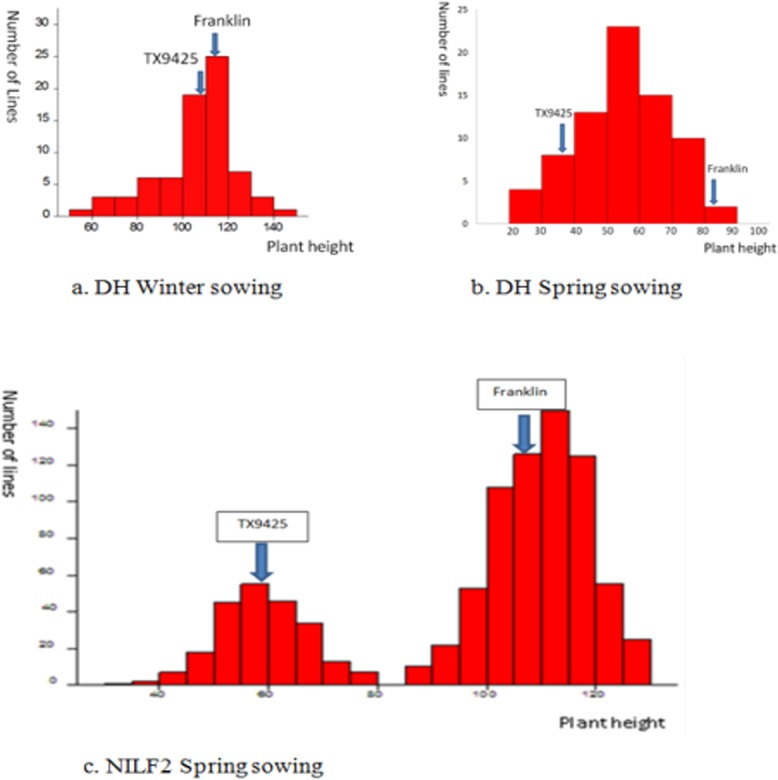
Distribution of plant height in the DH and NILF2 populations. (a) Continuous normal distribution of plant height for the 92 DH lines used in the winter-sown trial; (b) continuous bi-modal distribution of plant height for the 92 DH lines used in the spring-sown trial; (c) height distribution of 903 NILF_2_ plants showing two distinct groups.

The average height of the lines in the ‘TX9425/Franklin’ DH population varied from 58 to 143 cm in the winter sown trial in Launceston, Tasmania, and from 25.25 to 83.75 cm in the spring sown trial in Gatton, Queensland; all data were normally distributed (Fig. 2). The characteristics of these phenotypic data suggest that more than one gene control height.

The 269 F_8_ NIL plants were separated into three genotypic groups, based on the polymorphism of the SSR marker closely linked to the dwarfing QTL from ‘TX9425’. The first group was homozygous for the ‘TX9425’ allele, the second homozygous for the ‘Franklin’ allele, and the third heterozygous. Plant height in the ‘TX9425’ group ranged from 45 to 70 cm, averaging 65 cm. The average height of plants in the ‘Franklin’ group was 105 cm, with values ranging from 95 to 120 cm. The average height of the heterozygous group was 105 cm, with values ranging from 95 to 120 cm. These data suggest that the ‘TX9425’ allele reduces plant height by nearly 40 cm. The segregation ratio of the ‘TX9425’ type, the hybrid group, and the ‘Franklin’-type was close to a 1:2:1 ratio, resulting in a 3:1 ratio for tall and short plants (Table 3).

**Table 3:** Segregation of dwarf and tall progenies in the DH and NILF2 populations.

Population	No. of tall plants	No. of short plants	Ratio (tall:short)	P value
‘TX9425/Franklin’F8 NIL lines	198	71	3:1	p<0.01
‘TX9425/Franklin’ NILF2 population	693	248	3:1	p<0.01

The average height of individuals in the large NILF_2_ fine mapping population varied from 30 to 130 cm, and the data exhibited a bimodal distribution (Fig. 2), suggesting that only one major gene controls plant height. This supports the hypothesis that *sdw1* and other dwarfing genes have been separated; thus, the fine mapping population segregated only for the locus derived from ‘TX9425’. The 3:1 segregation ratio between the tall and dwarf individuals of the NILF_2_ population suggests that the target semi-dwarf gene derived from ‘TX9425’ is recessive, as opposed to *sdw1*, which is dominant (Table 3).

### Fine mapping of the dwarfing locus in ‘TX9425’

**Fig 3:**
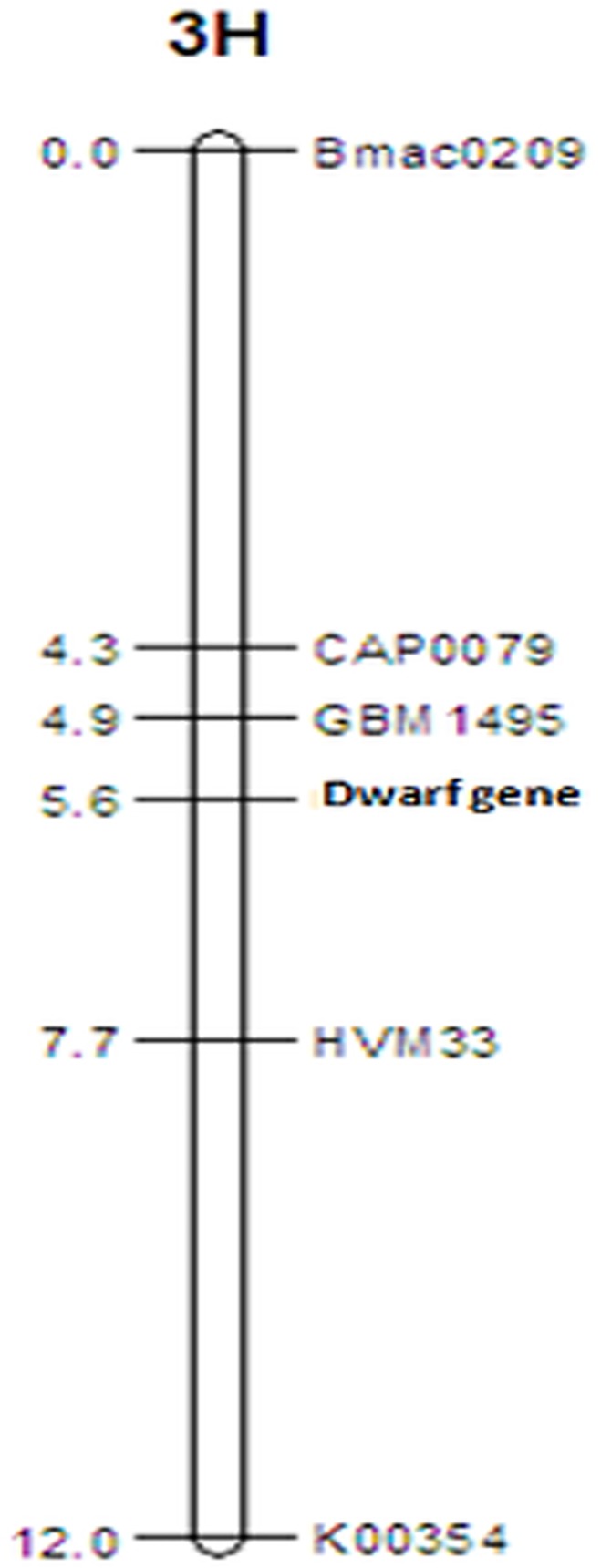
Fine mapping of the dwarfing locus derived from ‘TX9425’, a two-rowed Chinese landrace. The dwarfing gene was mapped to a 2.8 cM region, and was only 0.7 cM from the nearest marker, GBM1495.

To characterize the semi-dwarf gene in ‘TX9425’, a NILF_2_ fine mapping population segregating only for this locus was used to locate it precisely on chromosome 3H. We tested more than 50 sequence-tagged sites (STS) and CAPS markers from the region harboring the dwarfing QTL. Among them, 32 STS/CAPS markers were polymorphic between the tall and dwarf parents. Linkage analysis using 903 individuals segregating for the semi-dwarf locus localized the locus within a 2.9 cM region on chromosome 3HL, flanked by the markers HVM33 and GBM1495. The gene was 0.7 cM from GBM1495 and 2.1 cM from HVM33 (Fig. 3).

### Molecular cloning and sequence analysis of the ‘TX9425’ dwarfing gene

**Fig 4:**
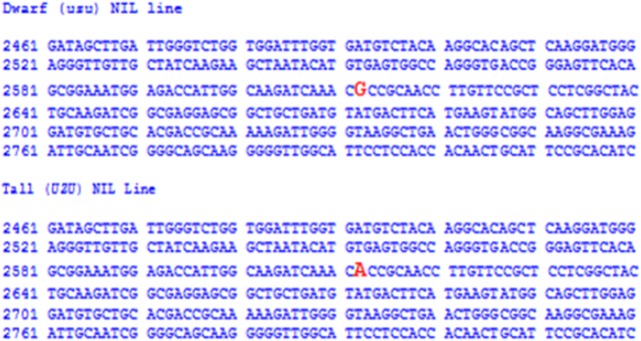
Sequences of the *HvBRI*1 homologs cloned from tall and dwarf NIL lines segregating only for the ‘TX9425’ semi-dwarfing locus. The comparison of the sequences identified the single base pair mutation from A to G at site 2612.

It has been reported that the *uzu* phenotype observed in six-rowed barley may be caused by single-nucleotide substitution (A to G) at position 2612 of *HvBRI1* [9]. To determine whether the semi-dwarf gene from ‘TX9425’ has one or several mutations in the *HvBRI1* sequence, a DNA fragment of the same size was amplified by end-to-end PCR from a set of NILs segregating for this locus, and then sequenced (S1 File). Sequence comparisons showed that the *HvBRI1* sequences in the tall and dwarf NILs are identical, except for a single nucleotide substitution (A-2612 to G-2612) in the semi-dwarf allele (Fig. 4), which led to the amino acid substitution of His (CAC) to Arg (CGC), as has been reported for the *uzu* allele in six-rowed barley [9].

**Fig 5:**
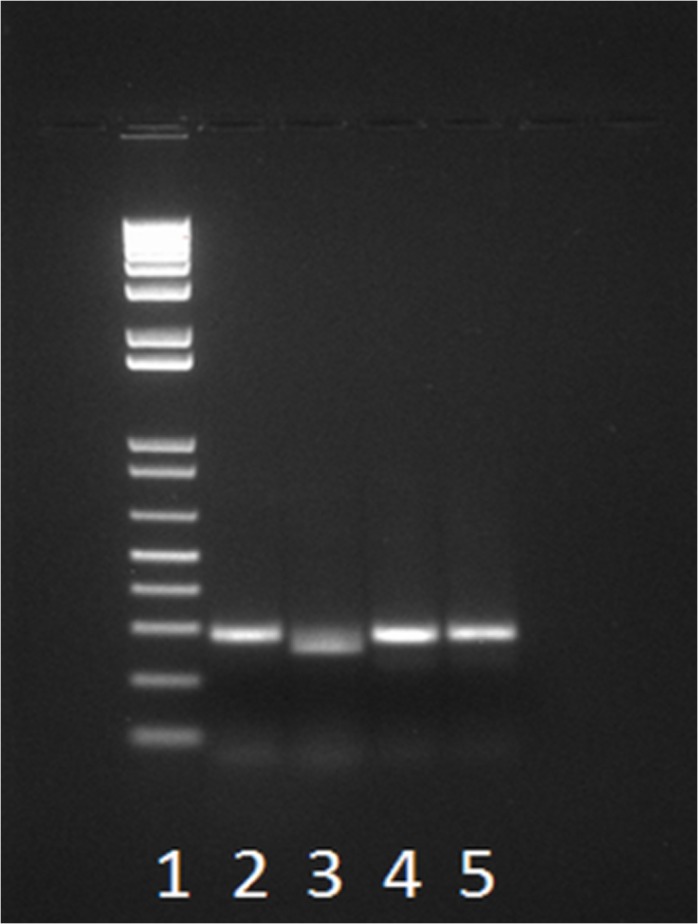
PCR amplification detecting the SNP difference between semi-dwarf and tall lines in a doubled haploid population segregating for the *uzu* locus. Lane 1: marker ladder; Lane 2: semi-dwarf (*uzu*), undigested; Lane 3: semi-dwarf (*uzu*), digested with *Hha*I; Lane 4: tall lines, undigested; Lane 5: tall lines, digested with *Hha*I.

**Fig 6:**
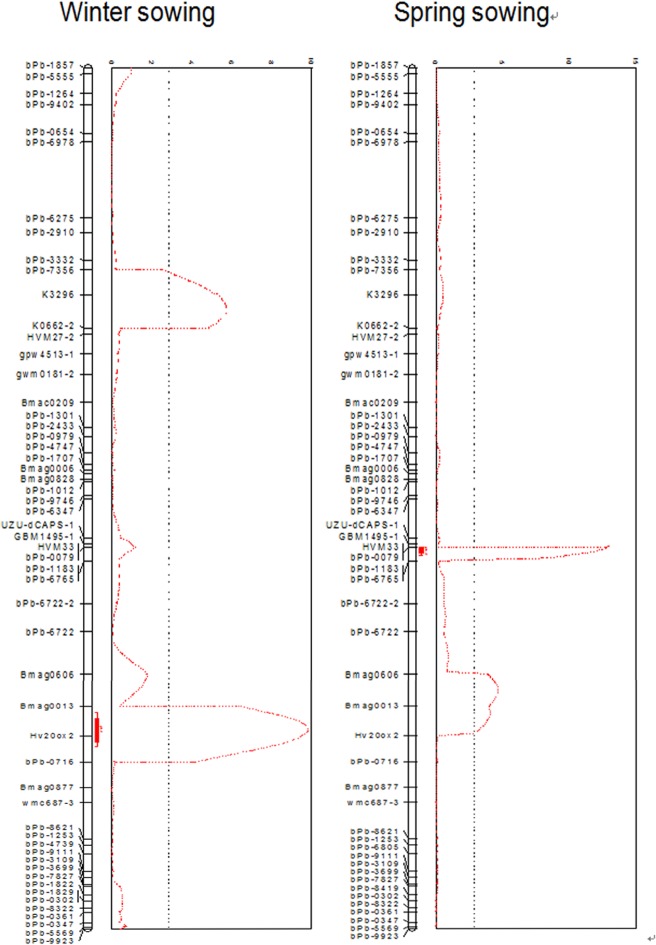
Diagnostic markers developed from the *uzu* and *sdw1* sequences were mapped on barley chromosome 3H. The linkage maps were constructed using Joinmap4.0, QTLs were detected in a composite interval mapping using MapQTL5.0. The diagnostic markers co-segregated with QTLs representing the dwarfing genes in the ‘TX9425/Franklin’ doubled haploid population, with a third QTL identified and located on chromosome 3HS.

In order to confirm the allelism between the *HvBRI1* SNP and the dwarfing gene isolated from ‘TX9425’, 92 DH lines derived from the ‘TX9425’ × ‘Franklin’ cross were used in the SNP analysis. We applied the dCAPS method to detect the SNPs between these two lines, with or without the semi-dwarf locus. In this study, the combination of constructed mismatch primer (dCAPS primer 1) and the second primer (dCAPS primer 2) created a specific recognition site for the restriction enzyme *Hha*I in the PCR product derived from the semi-dwarf lines (Fig. 5). In the DH population segregating for this semi-dwarfing gene, the SNP co-segregated with dwarfness in a 3:1 ratio, and the SNP was mapped to the same genomic region that harbored the semi-dwarfing QTL derived from ‘TX9425’ (Fig. 6). Therefore, the marker *dCAPSuzu* is ideal for the MAS of this dwarfing gene.

### Characterization of other dwarfing genes on barley chromosome 3H

To characterize the other dwarfing QTLs identified in the ‘TX9425/Franklin’ DH population, a previously identified diagnostic marker of *sdw1* (Jia et al., 2009), and three markers closely linked to the ‘TX9425’ semi-dwarf locus, were genotyped in the ‘TX9425/Franklin’ DH population. As shown in the resulting genetic map (Fig. 6), the *sdw1* diagnostic marker hv20ox2, co-segregating with the semi-dwarf QTL derived from ‘Franklin’, was located in a different region from that harboring the QTL representing the semi-dwarf locus derived from ‘TX9425’. The distance between the two genes was roughly 23 cM, with the ‘TX9425’ locus proximally located and *sdw1* distally located on chromosome 3HL. The third QTL, detected only in the winter-sown trials in this population, was located on chromosome 3HS at a distance of 21 cM from the ‘TX9425’ gene (Fig. 6). The markers HVM27, K0662 and K3296, which are closely linked to the presumably day length- and temperature-sensitive dwarfing QTL, can be used in the MAS of this gene.

## Discussion

### Characterization of the uzu allele derived from a two-rowed semi-dwarf barley landrace

Takahashi and Yamashi [8] have described in detail the typical characteristics of the *uzu* mutant, such as a short rachilla, short coleoptile, short glume and glume awn, short and wide leaves [33], dark leaf color, stiff culms, and erect leaves. These mutants resemble the wheat and rice varieties of the Green Revolution in that they are adapted to heavy manuring and dense field planting through a high harvest index and lodging resistance [8, 34].

The semi-dwarf phenotype of *uzu* barley may result from the reduced sensitivity of BRs encoded by *HvBRI1*, which has recently been identified as a barley homolog of *Arabidopsis BRI1* and of rice *D61* genes [9]. The sequence analysis of available BRI1 orthologs revealed that the cytoplasmic domains in BRI1 are conserved across species [35]. However, the extracellular domain of BRI1 is poorly conserved across species [35], and it has been reported that the extracellular region of *Arabidopsis* BRI1 is required for its full function [36]. Sequence comparisons of BRI1 homologs have shown that the rice and barley BRI1 proteins lack three LRR domains [9, 37], and their function as BR receptors remains to be ascertained. The evolutionary diversity of BRI1 among these species suggests that further analysis is needed to clarify the function of HvBRI1 in barley.

Studies of allelism in the *uzu* gene have been undertaken since the 1950s. Leonard et al. [38] and Garza-Falcon [39] claimed to have found two additional genes, *uzu2* and *uzu3*, based on the study of plant height alone [40]. However, there was no segregation of coleoptile characteristics in the F_2_ plants derived from crosses of the semi-dwarf genotypes carrying *uzu*, *uzu2*, and *uzu3*, suggesting that all three parental varieties contain the same *uzu* gene [41, 42]. Recently, Gruszka et al. [43] have developed a barley semi-dwarf mutant 093AR from mutagenic treatment of seeds of the cv. Aramir, and genetic analysis indicated that this chemically induced semi-dwarf gene was allelic to the spontaneous *uzu* [44]. A comparison of the genomic sequence of *HvBRI1* identified two CC>AA substitutions, at positions 1760 and 1761 in the barley *HvBRI1* gene of 093AR, that led to a missense mutation, causing a Thr-573>Lys-573 substitution in the extracellular domain of the BR receptor.

The two-rowed semi-dwarf barley landrace ‘TX9425’ used in this study exhibited typical phenotypic characteristics of *uzu* mutants. The 3:1 segregation of tall and dwarf lines in both the F_7_ and F_8_ generations of the NILs segregating only for the semi-dwarfing gene from ‘TX9425’ suggests that this mutation, like that in *uzu*, is recessive. Fine-mapping of this semi-dwarfing gene in a large NILF_2_ population containing 903 individuals located it within a region of 2.8 cM, flanked by the markers GBM1495 and HVM33 on chromosome 3HL. From the locations of these markers on the GrainGenes Barley bin map (http://wheat.pw.usda.gov), we have determined that the semi-dwarfing gene from ‘TX9425’ is located on 3H-bin7b. Molecular cloning and sequencing analyses showed that ‘TX9425’-derived semi-dwarfness is caused by a single-nucleotide substitution (A to G) at position 2612 of the *HvBRI1* gene, which is the same as the *uzu* mutation reported in six-rowed dwarf barley [9].

At least five independent loci controlling row type have been reported in barley; the six-rowed phenotype is controlled by the recessive gene *vrs1*, which is located on chromosome 2HL [45]. Other genes, such as *vrs2*, *vrs3*, *vrs4*, and *vrs5*, showed imperfect six-rowed phenotypes, and only *vrs4* was located in a similar genomic region as *uzu* on chromosome 3H. *vrs2*, *vrs3*, and *vrs5* were located on chromosomes 5H, 1H, and 4H respectively [46]. Therefore, in most cases *uzu* should segregate freely with the loci controlling row type in barley. However, studies on wild barley have suggested that the two-rowed spike is the ancestral form, which changed to six-rowed through mutation during domestication [45]. This suggests that the *uzu* mutation may be a single-mutation event that originated in two-rowed barley before the six-rowed mutation occurred during barley domestication, or that separate mutation events occurred after six-rowed barley mutated from its two-rowed ancestors.

### The effect of the semi-dwarf genes on barley chromosome 3H and molecular markers useful for MAS

In addition to the semi-dwarf gene *uzu*, there are other semi-dwarf genes located on barley chromosome 3H, including *sdw1*, *denso*, and possibly another gene [26, 47]. It has previously been reported that the *sdw1* allele reduced plant height by 10 to 20 cm [21], and Jia et al. [24] have reported a similar effect of the *denso* allele. The *sdw1* gene was allelic to the *denso* gene [17, 19, 20, 22], although it might involve a different mutation event [16].

Jia et al. [24] have proposed that *sdw1* is the most likely barley ortholog of *sd1* in rice, and they have identified GA-20 oxidase as a candidate for the semi-dwarf gene *sdw1*/*denso* in barley. The diagnostic SNP markers of *sdw1* were genotyped in the ‘TX9425/Franklin’ DH population in this study and co-localized with the peak of the dwarfing QTL derived from ‘Franklin’. The co-segregation of the *dCAPS/uzu* marker developed from the *HvBRI1* sequence and the semi-dwarfing QTL derived from ‘TX9425’ was confirmed by linkage analysis in this study. Therefore, the two major QTLs identified in this population represent the semi-dwarfing genes *uzu* and *sdw*1, and both are located on chromosome 3HL.

A third QTL identified on chromosome 3HS in this study was located 21 cM away from the *uzu* gene. This QTL was also derived from ‘TX9425’ and was only detected in the winter-sown trials, suggesting that it is temperature- and/or day-length sensitive. The discovery of the second gene (with a small effect) derived from ‘TX9425’ might explain why ‘TX9425’ performance is significantly different under winter- and spring-sown conditions. This is because the *uzu* gene was activated only in the spring season, in conditions of warm temperatures and long days (Table 2; Fig. 6), whereas the 3HS gene was active in the winter season, and had a much smaller effect than that of *uzu*. However, the difference in the height of ‘Franklin’ plants grown under spring and winter conditions is not as significant as that of ‘TX9425’ plants, perhaps because *sdw*1 is expressed in both seasons, albeit with a lesser effect in winter (Table 2).

When *uzu* and *sdw1* were expressed in the combined genetic background of ‘TX9425’/‘Franklin’, both of the two genes contributed to the reduction in plant height during the spring season. However, the expression of *uzu* was somewhat dominant over that of *sdw1* (Fig. 6), as the effect of *sdw1* in spring was less than that in winter, which is the opposite of the performance of this gene in the parental line, ‘Franklin’. Therefore, the interaction between *uzu* and *sdw1* is quite complex, and requires further investigation.

The semi-dwarfing cultivars carrying *uzu* exhibited improved lodging resistance and a high harvest index. However, more breeding or molecular mapping experiments are needed to evaluate the performance of semi-dwarf barley lines in terms of yield and yield-related traits and to understand the mechanisms underlying their growth regulation. For example, the effect of the reduction of coleoptile length by *uzu* [12, 48] on the seed-sowing angle in a drought environment needs to be assessed. Further studies are also needed to combine different dwarfing genes into the same background, and to efficiently investigate the advantages and disadvantages of using these genes in barley breeding programs. For instance, the *sdw1* semi-dwarf gene does not confer short coleoptile length [48], and it may alleviate the reduction of coleoptile length by *uzu*, if these two genes are pyramided in barley varieties.

## Conclusion

A barley semi-dwarfing gene, derived from the two-rowed Chinese landrace ‘TX9425’ was fine mapped to a 2.8 cM region close to the centromere on chromosome 3H, and a candidate gene for this locus, *HvBRI1*, was cloned in this study. Sequence analyses showed that the dwarfing landrace ‘TX9425’ contains a single nucleotide substitution of A to G at position 2612 in the *HvBRI1* gene, in accordance with a previous report of this mutation on this gene in six-rowed barley. The phenotypic characters of the ‘TX9425’-derived semi-dwarf locus, and its fine mapped location in the barley genome, suggest that this locus is same as the semi-dwarf *uzu* gene in six rowed barley. However, it is not clear whether the *uzu* mutation originated in two-rowed barley as a single-mutation event, before six-rowed barley arose from mutations in its two-rowed ancestors, or originated in two-rowed and six-rowed barley separately, following the six-rowed mutation during barley domestication.

The interaction of the ‘TX9425’-derived *uzu* gene with the other 3H semi-dwarfing gene, *sdw1*, was investigated in this study by expressing these genes in the same genetic background of ‘TX9425/Franklin’. Both these two genes contributed to the reduction in plant height in warm temperatures and during long days, and the expression of *uzu* was somewhat dominant over that of *sdw1*. These results have improved our understanding of the genetic mechanisms controlling the semi-dwarfness in barley, and the findings provide diagnostic markers for the selection of genes for semi-dwarf stature in barley breeding programs.

## Supporting Information

S1 File Full sequences of the *HvBR1* alleles isolated from the dwarf and tall two-rowed *uzu* near iso-genic lines. (I) full sequence of the dwarf NIL line; (II) full sequence of the tall NIL line.
(PDF)
