# Supplementary material for: Molecular Characterization of Barley 3H Semi-Dwarf Genes
Source: PLoS One. 2015 Mar 31;10(3):e0120558. doi: 10.1371/journal.pone.0120558 (PMC4380412; doi:10.1371/journal.pone.0120558)
Supplement: S1 File — (I) full sequence of the dwarf NIL line; (II) full sequence of the tall NIL line. (PDF) [file pone.0120558.s001.pdf]

(I) full sequence of the dwarf NIL line

```
1       CGCTTCTCGC ATGGTCTCAA GGTAGAGCGA GCGAGCTCCC ACATGGATTG CCTGCGGCTG
61      GCGGTAGCGG CGGCGGCGCT CCTTCTCGCG GCGCTCGCGG CGGCGGCCGA CGACGCGCAG
121     CTGCTGGACG AGTTCAGGAT GGCGCTGCCC AGCCAGGCGC CGCTCGAAGG CTGGACCGCG
181     CGGGAGGGCG CCTGCAGGTT CCCCGGCGCG GTGTGCCGGG GCGGGCGGCT CACGTCGCTC
241     TCGCTCGCCG CCGTCACGCT CAATGCCGAC TTCCGCGCCG TCGCCAACAC CCTGCTGCAG
301     CTCAGCGCCG TCGAGCGGCT CAGCCTGCGC GGCGCCAACG TCAGCGGCGC GCTCGCGGCC
361     GCCAGGTGCG GCGGCAAGCT CGAGGAGCTC GACCTCTCCG GGAATGCCGC GCTGCGCGGC
421     TCCGTCGCCG ACGTCGCCGC GCTCGCCGGC TCCTGCGGCG CGCTCCGGAC GCTGAATCTC
481     TCCGGTGACG CGGTTGGTGC GGCCAAGCCC GCTGGCGGTG GCGGTGGCGG GCAGGGGTTT
541     GCGGCCCTCG ACGCCCTCGA CTTGTCCAGC AACAAGATCG CCGGAGATGC CGACCTTCGC
601     TGGATGGTGG GTGCCGGCCT TGGGTCTGTC CGGTGGCTGG ACCTCGCCTG GAACAAGATC
661     TCCGGCGGCC TCTCGGACTT CACCAACTGC TCCGGCCTGC AGTACCTTGA CCTATCCGGC
721     AACCTCATCG CCGGCGATGT CGCCGCGGCG GCGCTCTCCG GCTGCCGCAG CCTCAGAGCG
781     CTCAACCTCT CCAGCAACCA TCTCGCCGGC GCGTTCCCGC CCAACATCGC CGGCCTCACC
841     TCGCTCACCG CCCTCAACCT GTCCAACAAC AACTTCTCTG GCGAGGTCCC CGCCGACGCT
901     TTCACCGGCC TGCAGCAGCT CCAGTCGCTG TCCCTCTCCT TCAACCACTT CAGCGGCTCC
961     ATCCCTGATT CCGTCGCTGC GCTGCCGGAC CTCGAGGTGC TCGACCTCAG CTCCAACAAC
1021    TTCTCCGGCA GCATCCCCGA CTCCCTCTGC CAAGATCCCA ACTCGAGGCT CCGCGTGCTC
1081    TACCTTCAGA ACAACTATCT CTCCGGCAGC ATCCCGGAGG CCGTCTCCAA CTGCACCGAC
1141    CTCGTCTCCC TCGACCTCAG TCTCAACTAC ATCAACGGCT CCATCCCGGA ATCCCTCGGC
1201    GAGCTTAGCC GCCTCCAGGA CCTTATCATG TGGCAGAACT TACTGGAGGG CGAGATACCT
1261    GCGTCTCTGT CAAGCATTCC CGGCCTGGAG CATCTCATCC TCGACTACAA CGGGCTCACC
1321    GGCAGCATCC CGCCGGAGCT GGCCAAGTGC AAGCAGCTCA ACTGGATATC CTTGGCGAGC
1381    AACCGGCTGT CTGGGCCGAT CCCCTCGTGG CTTGGGAAGC TCAGCAACTT GGCAATCCTA
1441    AAGCTGAGCA ATAATTCCTT CACCGGAAAG ATACCCGCAG AGCTTGGTGA CTGCAAGAGC
1501    TTGGTATGGC TGGACCTCAA CAGCAACCAA CTTAATGGAT CAATTCCACC CGAGCTGGCA
1561    GAGCAGTCGG GGAAGATGAC CGTTGGCCTC ATTATTGGGC GGCCCTATGT GTATCTTCGC
1621    AACGACGAGC TGAGCAGCCA GTGTCGTGGC AAGGGCAGCT TGCTGGAGTT CTCAAGCATC
1681    CGGTCCGAGG ACCTTAGTCG GATGCCGAGC AAGAAGCTGT GCAACTTCAC AAGGATGTAC
1741    ATGGGGAGCA CAGAGTATAC CTTCAACAAG AATGGCTCCA TGATATTTCT GGATTTGTCA
1801    TTTAATCAGC TTGACTCGGA GATACCCAAG GAGCTTGGCA ATATGTTCTA CCTCATGATC
1861    ATGAATCTTG GGCACAACCT TCTGTCTGGT GCTATCCCAA CAGAGCTAGC TGGTGCAAAG
1921    AAGCTTGCAG TACTTGACCT TTCGCACAAC CGATTGGAAG GGCAGATACC CAGCTCGTTC
1981    TCGTCACTGT CTTTGTCTGA GATCAATCTG TCGAGTAATC AGCTGAATGG GACAATTCCA
2041    GAGCTTGGTT CTCTTGCCAC ATTCCCAAAG AGCCAGTATG AGAACAACTC CGGTCTGTGT
2101    GGCTTTCCAC TGCCACCATG TGAGTCGCAT ACTGGGCAAG GCTCTTCCAA CGGTGGCCAA
2161    TCTAACCGGA GGAAGGCGTC CCTTGCGGGC AGCGTTGCTA TGGGACTCTT GTTCTCGCTC
2221    TTCTGTATAT TTGGGTTGGT CATCATAGCC ATCGAGAGCA AGAAGCGGAG GCAGAAGAAT
2281    GACGAGGCAA GTACCTCCCG TGATATATAC ATTGATAGCC GGTCACATTC TGGCACGATG
2341    AATTCCAATT GGAGACTCTC TGGTACAAAT GCTCTCAGCA TCAACCTTGC TGCATTTGAG
2401    AAGCCACTGC AGAAACTCAC CTTGGGTGAT CTTGTTGAGG CCACCAATGG CTTCCACAAT
2461    GATAGCTTGA TTGGGTCTGG TGGATTTGGT GATGTCTACA AGGCACAGCT CAAGGATGGG
2521    AGGGTTGTTG CTATCAAGAA GCTAATACAT GTGAGTGGCC AGGGTGACCG GGAGTTCACA
2581    GCGGAAATGG AGACCATTGG CAAGATCAAA CGCCGCAACC TTGTTCCGCT CCTCGGCTAC
2641    TGCAAGATCG GCGAGGAGCG GCTGCTGATG TATGACTTCA TGAAGTATGG CAGCTTGGAG
2701    GATGTGCTGC ACGACCGCAA AAAGATTGGG GTAAGGCTGA ACTGGGCGGC AAGGCGAAAG
2761    ATTGCAATCG GGGCAGCAAG GGGGTTGGCA TTCCTCCACC ACAACTGCAT TCCGCACATC
2821    ATCCACCGAG ACATGAAGTC AAGCAATGTG CTTGTTGATG AGAATCTGGA GGCAAGGGTC
2881    TCTGATTTCG GCATGGCGAG GATGATGAGT GTGGTGGACA CACACCTGAG CGTGTCCACC
2941    CTCGCCGGCA CGCCGGGGTA TGTGCCACCG GAGTACTACC AGAGCTTCCG ATGCACCACC
3001    AAGGGTGATG TGTATAGCTA CGGTGTTGTG TTGCTGGAGC TGCTCACTGG GAAGCCCCCG
3061    ACGGATTCAA CAGATTTTGG TGAGGACCAT AATCTTGTAG GATGGGTGAA GATGCACACA
3121    AAGTTGAAGA TCACTGATGT GTTTGACCCG GAGCTGCTGA AGGACGATCC CACCCTGGAG
3181    CTTGAGCTGC TGGAGCATTT GAAAATCGCA TGTGCGTGCC TGGATGACAG GCCGTCGAGG
3241    CGACCAACGA TGCTCAAGGT GATGACGATG TTCAAGGAGA TCCAGGCTGG GTCAACGGTG
3301    GACTCGAAAA CCTCGTCGGT GGCTACCGGG CTGAGCGACG ATCCTGGTTT TGGGGTCATG
3361    GACATGACCC TCAAGGAGGC CAAGGAGGAG AAAGATTAGG AAGATGACCA CCGGCGACAC
3421    GCAGACGGCG GTGACCGGCA GCAACACGTC CGTCCGTCCG TCCAGTTTGG GTTTGGGGGC
3481    GAAAAAACCC GTAGAAATCA GCCTCTCAGA TGATTGATGA TGATGTGATG ATACCATTCT
3541    GAGGTTCCCA CGCAAGCT
```

(II) full sequence of the tall NIL line

```
1       CGCTTCTCGC ATGGTCTCAA GGTAGAGCGA GCGAGCTCCC ACATGGATTG CCTGCGGCTG
61      GCGGTAGCGG CGGCGGCGCT CCTTCTCGCG GCGCTCGCGG CGGCGGCCGA CGACGCGCAG
121     CTGCTGGACG AGTTCAGGAT GGCGCTGCCC AGCCAGGCGC CGCTCGAAGG CTGGACCGCG
181     CGGGAGGGCG CCTGCAGGTT CCCCGGCGCG GTGTGCCGGG GCGGGCGGCT CACGTCGCTC
241     TCGCTCGCCG CCGTCACGCT CAATGCCGAC TTCCGCGCCG TCGCCAACAC CCTGCTGCAG
301     CTCAGCGCCG TCGAGCGGCT CAGCCTGCGC GGCGCCAACG TCAGCGGCGC GCTCGCGGCC
361     GCCAGGTGCG GCGGCAAGCT CGAGGAGCTC GACCTCTCCG GGAATGCCGC GCTGCGCGGC
421     TCCGTCGCCG ACGTCGCCGC GCTCGCCGGC TCCTGCGGCG CGCTCCGGAC GCTGAATCTC
481     TCCGGTGACG CGGTTGGTGC GGCCAAGCCC GCTGGCGGTG GCGGTGGCGG GCAGGGGTTT
541     GCGGCCCTCG ACGCCCTCGA CTTGTCCAGC AACAAGATCG CCGGAGATGC CGACCTTCGC
601     TGGATGGTGG GTGCCGGCCT TGGGTCTGTC CGGTGGCTGG ACCTCGCCTG GAACAAGATC
661     TCCGGCGGCC TCTCGGACTT CACCAACTGC TCCGGCCTGC AGTACCTTGA CCTATCCGGC
721     AACCTCATCG CCGGCGATGT CGCCGCGGCG GCGCTCTCCG GCTGCCGCAG CCTCAGAGCG
781     CTCAACCTCT CCAGCAACCA TCTCGCCGGC GCGTTCCCGC CCAACATCGC CGGCCTCACC
841     TCGCTCACCG CCCTCAACCT GTCCAACAAC AACTTCTCTG GCGAGGTCCC CGCCGACGCT
901     TTCACCGGCC TGCAGCAGCT CCAGTCGCTG TCCCTCTCCT TCAACCACTT CAGCGGCTCC
961     ATCCCTGATT CCGTCGCTGC GCTGCCGGAC CTCGAGGTGC TCGACCTCAG CTCCAACAAC
1021    TTCTCCGGCA GCATCCCCGA CTCCCTCTGC CAAGATCCCA ACTCGAGGCT CCGCGTGCTC
1081    TACCTTCAGA ACAACTATCT CTCCGGCAGC ATCCCGGAGG CCGTCTCCAA CTGCACCGAC
1141    CTCGTCTCCC TCGACCTCAG TCTCAACTAC ATCAACGGCT CCATCCCGGA ATCCCTCGGC
1201    GAGCTTAGCC GCCTCCAGGA CCTTATCATG TGGCAGAACT TACTGGAGGG CGAGATACCT
1261    GCGTCTCTGT CAAGCATTCC CGGCCTGGAG CATCTCATCC TCGACTACAA CGGGCTCACC
1321    GGCAGCATCC CGCCGGAGCT GGCCAAGTGC AAGCAGCTCA ACTGGATATC CTTGGCGAGC
1381    AACCGGCTGT CTGGGCCGAT CCCCTCGTGG CTTGGGAAGC TCAGCAACTT GGCAATCCTA
1441    AAGCTGAGCA ATAATTCCTT CACCGGAAAG ATACCCGCAG AGCTTGGTGA CTGCAAGAGC
1501    TTGGTATGGC TGGACCTCAA CAGCAACCAA CTTAATGGAT CAATTCCACC CGAGCTGGCA
1561    GAGCAGTCGG GGAAGATGAC CGTTGGCCTC ATTATTGGGC GGCCCTATGT GTATCTTCGC
1621    AACGACGAGC TGAGCAGCCA GTGTCGTGGC AAGGGCAGCT TGCTGGAGTT CTCAAGCATC
1681    CGGTCCGAGG ACCTTAGTCG GATGCCGAGC AAGAAGCTGT GCAACTTCAC AAGGATGTAC
1741    ATGGGGAGCA CAGAGTATAC CTTCAACAAG AATGGCTCCA TGATATTTCT GGATTTGTCA
1801    TTTAATCAGC TTGACTCGGA GATACCCAAG GAGCTTGGCA ATATGTTCTA CCTCATGATC
1861    ATGAATCTTG GGCACAACCT TCTGTCTGGT GCTATCCCAA CAGAGCTAGC TGGTGCAAAG
1921    AAGCTTGCAG TACTTGACCT TTCGCACAAC CGATTGGAAG GGCAGATACC CAGCTCGTTC
1981    TCGTCACTGT CTTTGTCTGA GATCAATCTG TCGAGTAATC AGCTGAATGG GACAATTCCA
2041    GAGCTTGGTT CTCTTGCCAC ATTCCCAAAG AGCCAGTATG AGAACAACTC CGGTCTGTGT
2101    GGCTTTCCAC TGCCACCATG TGAGTCGCAT ACTGGGCAAG GCTCTTCCAA CGGTGGCCAA
2161    TCTAACCGGA GGAAGGCGTC CCTTGCGGGC AGCGTTGCTA TGGGACTCTT GTTCTCGCTC
2221    TTCTGTATAT TTGGGTTGGT CATCATAGCC ATCGAGAGCA AGAAGCGGAG GCAGAAGAAT
2281    GACGAGGCAA GTACCTCCCG TGATATATAC ATTGATAGCC GGTCACATTC TGGCACGATG
2341    AATTCCAATT GGAGACTCTC TGGTACAAAT GCTCTCAGCA TCAACCTTGC TGCATTTGAG
2401    AAGCCACTGC AGAAACTCAC CTTGGGTGAT CTTGTTGAGG CCACCAATGG CTTCCACAAT
2461    GATAGCTTGA TTGGGTCTGG TGGATTTGGT GATGTCTACA AGGCACAGCT CAAGGATGGG
2521    AGGGTTGTTG CTATCAAGAA GCTAATACAT GTGAGTGGCC AGGGTGACCG GGAGTTCACA
2581    GCGGAAATGG AGACCATTGG CAAGATCAAA CACCGCAACC TTGTTCCGCT CCTCGGCTAC
2641    TGCAAGATCG GCGAGGAGCG GCTGCTGATG TATGACTTCA TGAAGTATGG CAGCTTGGAG
2701    GATGTGCTGC ACGACCGCAA AAAGATTGGG GTAAGGCTGA ACTGGGCGGC AAGGCGAAAG
2761    ATTGCAATCG GGGCAGCAAG GGGGTTGGCA TTCCTCCACC ACAACTGCAT TCCGCACATC
2821    ATCCACCGAG ACATGAAGTC AAGCAATGTG CTTGTTGATG AGAATCTGGA GGCAAGGGTC
2881    TCTGATTTCG GCATGGCGAG GATGATGAGT GTGGTGGACA CACACCTGAG CGTGTCCACC
2941    CTCGCCGGCA CGCCGGGGTA TGTGCCACCG GAGTACTACC AGAGCTTCCG ATGCACCACC
3001    AAGGGTGATG TGTATAGCTA CGGTGTTGTG TTGCTGGAGC TGCTCACTGG GAAGCCCCCG
3061    ACGGATTCAA CAGATTTTGG TGAGGACCAT AATCTTGTAG GATGGGTGAA GATGCACACA
3121    AAGTTGAAGA TCACTGATGT GTTTGACCCG GAGCTGCTGA AGGACGATCC CACCCTGGAG
3181    CTTGAGCTGC TGGAGCATTT GAAAATCGCA TGTGCGTGCC TGGATGACAG GCCGTCGAGG
3241    CGACCAACGA TGCTCAAGGT GATGACGATG TTCAAGGAGA TCCAGGCTGG GTCAACGGTG
3301    GACTCGAAAA CCTCGTCGGT GGCTACCGGG CTGAGCGACG ATCCTGGTTT TGGGGTCATG
3361    GACATGACCC TCAAGGAGGC CAAGGAGGAG AAAGATTAGG AAGATGACCA CCGGCGACAC
3421    GCAGACGGCG GTGACCGGCA GCAACACGTC CGTCCGTCCG TCCAGTTTGG GTTTGGGGGC
3481    GAAAAAACCC GTAGAAATCA GCCTCTCAGA TGATTGATGA TGATGTGATG ATACCATTCT
3541    GAGGTTCCCA CGCAAGCT
```
